# The Infant to School programme: supporting school readiness in children and developing community nursery nurses within health visiting teams

**DOI:** 10.1093/pubmed/fdaf155

**Published:** 2025-12-06

**Authors:** Sharin Baldwin, Liza Azizpoor, Marzia Keshani, Wendy Sumpton, Marie McLouglin, Kathy Donohoe, Lynn Kemp

**Affiliations:** Innovation and Research, Institute of Health Visiting, c/o Royal Society for Public Health, John Snow House, 59 Mansell Street, London E1 8AN, UK; School of Nursing and Midwifery, Western Sydney University, Locked Bag 1797, Penrith, NSW 2751, Australia; Centre for Health Services Studies, University of Kent, Canterbury, Kent CT2 7NZ, UK; Central London Community Health Care Trust – Brent, Sudbury Primary Care, Vale Farm, Watford Road, Wembley, Middlesex HA0 3HG, UK; Central London Community Healthcare NHS Trust – Brent, Willesden Centre for Health & Care, Robson Avenue, London, NW10 3RY, UK; School of Nursing and Midwifery, Western Sydney University, Locked Bag 1797, Penrith, NSW 2751, Australia; Brent Council, Brent Civic Centre, Engineers Way, Wembley, London, HA9 0FJ, UK; Centre for Translational Research and Social Innovation (TReSI), School of Nursing and Midwifery, Western Sydney University, Locked Bag 1797, Penrith, NSW 2751, Australia; Ingham Institute for Applied Medical Research, 1 Campbell St, Liverpool, NSW 2170, Australia; Centre for Translational Research and Social Innovation (TReSI), School of Nursing and Midwifery, Western Sydney University, Locked Bag 1797, Penrith, NSW 2751, Australia; Ingham Institute for Applied Medical Research, 1 Campbell St, Liverpool, NSW 2170, Australia

**Keywords:** community nursery nurses, early intervention, health promotion programme, health visiting skill mix, school readiness

## Abstract

**Background:**

Community nursery nurses (CNNs) play a vital role in UK health visiting teams, promoting child development and school readiness. The Infant to School (I2S) programme, delivered by CNNs under health visitor supervision, provides structured early intervention for families facing adversity.

**Aim:**

To formatively evaluate the I2S programme from the perspective of CNNs, focusing on short-term outcomes, reported effects on children and families, and impacts on CNNs.

**Methods:**

Seventeen of twenty CNNs (85%) completed an anonymised questionnaire. Quantitative data were analysed descriptively, and qualitative responses were analysed thematically.

**Findings:**

Between September 2023 and February 2025, 212 families participated in the I2S programme, with language development as the main concern. CNNs reported that I2S enhanced their confidence, skills, and job satisfaction, enabling more structured, culturally sensitive, and relationship-based support. All respondents reported helping families to set and achieve short-term goals and connect with community services; 88% reported building strong relationships. Key themes included improved professional competence, greater ability to support families, and identified areas for further training and resource development.

**Conclusion:**

This evaluation contributes new insight into the role of CNNs in supporting school readiness through a structured, health visiting-embedded programme. Continued evaluation, incorporating parental and child outcome data, is required to assess long-term impact and scalability.

## Background

Health visitors in the UK are Public Health Nurses who deliver the Healthy Child Programme, supporting families from pregnancy until children start school. Most health visiting services use a skill-mix model, ‘a combination of staff and skills within a team who work together to optimise health outcomes’.^1^ This includes community nursery nurses (CNNs), who promote health (e.g. breastfeeding, nutrition, play, and parenting) and support child development (health reviews and behaviour management). Their role focuses on child development, behaviour, and school readiness, making them a vital part of the team to help children get the best start in life.

A 2023 survey of 1392 practitioners by the Institute of Health Visiting found that 84% reported increased demand for services, with child behaviour problems the second most common issue in Scotland, Wales, and Northern Ireland.^2^ A review of UK public health systems and policy approaches to early child development also identified school readiness as a key public health concern.^3^ Assessed at age 4–5 across 12 areas (including literacy, numeracy, self-care, and relationships), school readiness has been negatively affected by the COVID-19 pandemic, which disrupted children’s speech, emotional regulation, and motor skills.^4^^,^^5^

A Kindred2 report, based on 1000 teachers and 1000 parents, showed many children lack basic skills when starting school: 39% struggled to hold a pencil, 38% had trouble playing with peers, and over a third could not dress themselves or follow instructions.^6^ Half of teachers reported more children were not school-ready in 2023 than in 2022, and 43% of parents were unaware of school readiness before age four.

An OECD/World Bank study involving 2578 school professionals found UK children were less prepared for school than their international peers in the USA, Netherlands, India, Brazil, and South Africa.^7^ Gaps in literacy, numeracy, and socio-emotional skills were linked to inequality, fragmented early years provision, and limited early years investment. Poor school readiness has long-term impacts on education and employment,^8^ reinforcing the need for early intervention.

Children’s development varies by gender, age, and socioeconomic status.^9–12^ By age 4–5, those from disadvantaged families lag an estimated five months behind their peers,^13^ with similar patterns seen in the USA^14^ and Ireland.^15^ Addressing these disparities is a UK Government priority under the Levelling Up agenda, but requires investment, targeted support, and collaboration between home and school.^16^ CNNs, as part of skill-mix teams, are well placed to identify needs early, prevent escalation, and connect families with services.^17^

The Infant 2 School (I2S) programme is a unique, family-centred early intervention delivered by CNNs under health visitor supervision.^18^ To our knowledge, this programme is first of its kind in the UK. Based on the Maternal Early Childhood Home Visiting (MECSH) framework,^19^^,^^20^ it complements the health visiting offer and ensures timely, evidence-based support for vulnerable children.^21^

While previous work described the programme’s development and intended outcomes,^21^ this paper presents findings from an early-stage, formative evaluation of the I2S programme rollout in one London borough (Brent). It focuses on CNNs’ experiences and perspectives 1 year after implementation.

### School readiness in Brent

The London Borough of Brent is among the UK’s most ethnically diverse areas, with 65% of residents from minority ethnic backgrounds.^22^ Over a third of households experience disadvantage in income, employment, health, and housing.^23^ These challenges impact early development, especially speech and language skills, which are central to school readiness. Parents play a vital role in preparing children for Nursery/Foundation Stage 1, but Brent children often perform below London and national averages on school readiness indicators (Table 1).

**Table 1:** School readiness markers for Brent, London, and England (source: Fingertips | Department of Health and Social Care).

School Readiness Marker	Brent	London	England
Percentage of children achieving a good level of development at the end of Reception	65.7	67.8	65.2
Percentage of children achieving at least the expected level in communication and language skills at the end of Reception	77.1	79.1	79.5
Percentage of children achieving the expected level in the phonics screening check in Year 1	72.3	78.2	75.5

A key component of early identification and support for parents is the early education offer, which provides free nursery provision for vulnerable 2-year-olds, available to families receiving certain benefits. Eligible children receive 15 hours of free education weekly. However, uptake in Brent has been low: 49% in 2021, improving to 56.7% in 2022—still below London and national averages. For 3–4-year-olds, uptake is higher but remains lower than elsewhere (Table 2). Expanding access is crucial to improving outcomes in communication, language, and overall readiness.

**Table 2:** Percentage of children taking up funded placement in nursery. (source: London borough of Brent – Child and Young People’s Department).

Area	2021		2022		2023	
	2-year-olds	3 and 4-year-olds	2-year-olds	3 and 4-year-olds	2-year-olds	3 and 4-year-olds
Brent (%)	49	71.3	56.7	77.3	65.4	79.3
London (%)	50.2	80.3	62	82.3	65.2	83.7
England (%)	61.8	89.7	71.9	92.3	73.9	93.7

### Implementation of the I2S programme in Brent

I2S launched in Brent in September 2023. To support the delivery of the programme, CNN capacity within health visiting teams was doubled, from 10 to 20, with each CNN expected to support 20–25 families, devoting half their time to I2S and half to other delegated health visiting tasks. A Senior Nursery Nurse role was created to oversee training, supervision, and case allocation.

All CNNs completed three mandatory MECSH e-learning modules (plus three optional), followed by 2 days’ MECSH Foundation training and 1 day I2S training, also attended by health visitors. Eighteen of twenty CNNs completed training.

Once trained, the CNNs received referrals for the I2S programme, following an initial assessment conducted by the case-holding health visitor. Families were eligible if facing sustained adversity, including: multi-agency health/social needs, limited parenting capacity, and poor service engagement.

Families were required to stay on the programme for at least 12 months, receiving a minimum of four visits annually, with more provided as needed, as per the I2S programme specification.^9^

### Study aim

To conduct a formative evaluation of the I2S programme from the perspective of CNNs, focusing on short-term outcomes, reported effects on children and families, and impacts on CNNs.

## Methods

During routine I2S enrolment, CNNs collect standardised demographic and health data for each child, which are stored in a central database (Australia). A Senior Research Officer (K.D.) conducted analysis of this data.

To explore the experiences and perceptions of CNNs delivering the I2S programme, a bespoke anonymous questionnaire was developed. Although not formally validated, the tool was co-designed by the UK research lead (S.B.) in collaboration with the I2S Lead (L.A.), MECSH Advisor (M.K.), and MECSH Consultant (W.S.) to ensure face validity, alignment with the I2S theory of change, and clarity of questions. The questionnaire was piloted with two CNNs for readability and usability before wider dissemination. It addressed: outcomes anticipated in the theory of change (Box 1), and practitioner experience (barriers, facilitators, resources, job satisfaction, and training needs).

Box 1 I2S short-term outcomes: 6–12 months from programme start.

- Parents and practitioners build good working relationships

- Parents commit to I2S

- Parents experience a positive transition to parenting their new child

- Parents have information and support they need to meet immediate parenting needs and goals (0–6 weeks)

- Parents identify and achieve short term aims for the child, themselves, and their family (7–12 weeks)

- Parents identify and achieve longer term aims for the child, themselves, and their family (13–52 weeks)

- Parents start building supportive community links and informal/formal support

- Practitioners delivering I2S feel supported themselves

- Relevant practitioners and partner agencies understand and support the programme

As this was a service evaluation, formal ethics approval was not required. Participation was voluntary, anonymous, and unrelated to performance appraisal. To minimise bias, the questionnaire was distributed by the I2S Lead (L.A.) but returned anonymously to S.B., an independent researcher.

At the time of this early evaluation, most families had not yet completed the I2S programme, and fewer than 10% had reached all three assessment points of the validated outcome measures [Ages and Stages Questionnaire, Third Edition (ASQ-3), Parental Responsiveness Rating Scale (PaRRiS) Home Observation Measurement of the Environment Short Form (HOME-SF)]. As such, robust statistical comparisons across baseline, midpoint, and endpoint data were not feasible. These tools were nevertheless embedded into the programme’s data collection framework to establish consistency and ensure that future longitudinal analyses can be undertaken as more follow-up data become available.

Quantitative survey data were summarised using descriptive statistics (frequencies and percentages). Inferential statistical testing was not conducted due to the small sample size and the formative focus of the evaluation. This descriptive approach was considered proportionate to the study’s aim of generating early insights to refine programme delivery. Qualitative data were analysed thematically to identify key patterns and insights in practitioners’ experiences.

### Findings

**Figure 1:**
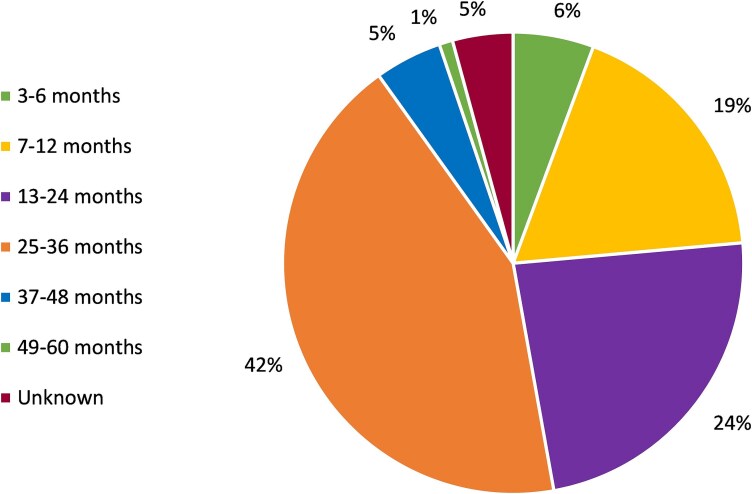
Child age at start of the I2S programme.

Between September 2023 and February 2025, 212 families were registered for I2S. The largest proportion of children was aged between 25 and 36 months (42%), followed by 13–24 months (24%), and 7–12 months (19%). A full breakdown of the age groups of the children is presented in Fig. 1. For families with multiple eligible children, the age of the youngest child is reported.

**Figure 2:**
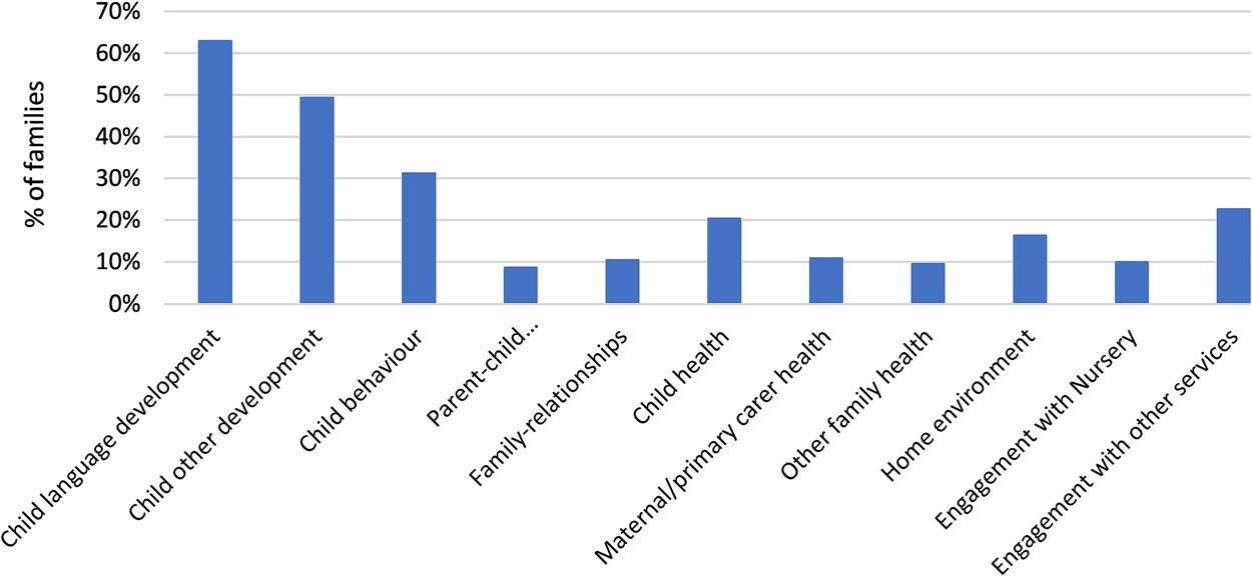
Parent/carer concerns and reasons for joining the I2S programme.

Families enrolled in the programme presented with a range of concerns, identified either by parents/carers or I2S practitioners. Most families (63%) reported concerns related to child language development. Other commonly identified areas included child development (49%), child behaviour (31%), engagement with other services (23%), child health (20%), home environment (16%), maternal or primary carer health (11%), and family relationships (10%) (see Fig. 2).

Exploratory analysis was conducted to examine potential relationships between child/family characteristics and the concerns recorded at enrolment. For example, language development was the most frequently reported concern across all age groups but was particularly common among children aged 25–36 months. Behavioural concerns were more often noted in the older age groups (37–60 months), whereas concerns relating to child health and maternal/primary carer health were distributed more evenly across the age spectrum. Although goal achievement data were not yet available in sufficient quantity to allow robust comparisons, these early patterns suggest that different developmental stages are associated with distinct family support needs.

A total of 17 out of 20 CNNs (85%) completed and submitted the anonymised questionnaire. Of the remaining three, two were on extended leave and one was not actively working with I2S families at the time of data collection and were therefore not eligible to provide informed feedback on programme implementation. This response rate represents near-complete participation from all eligible practitioners, minimising the risk of non-response bias. While self-selection bias cannot be entirely excluded, the sample is considered broadly representative of the practitioner group delivering the I2S programme in Brent during the study period.

**Figure 3:**
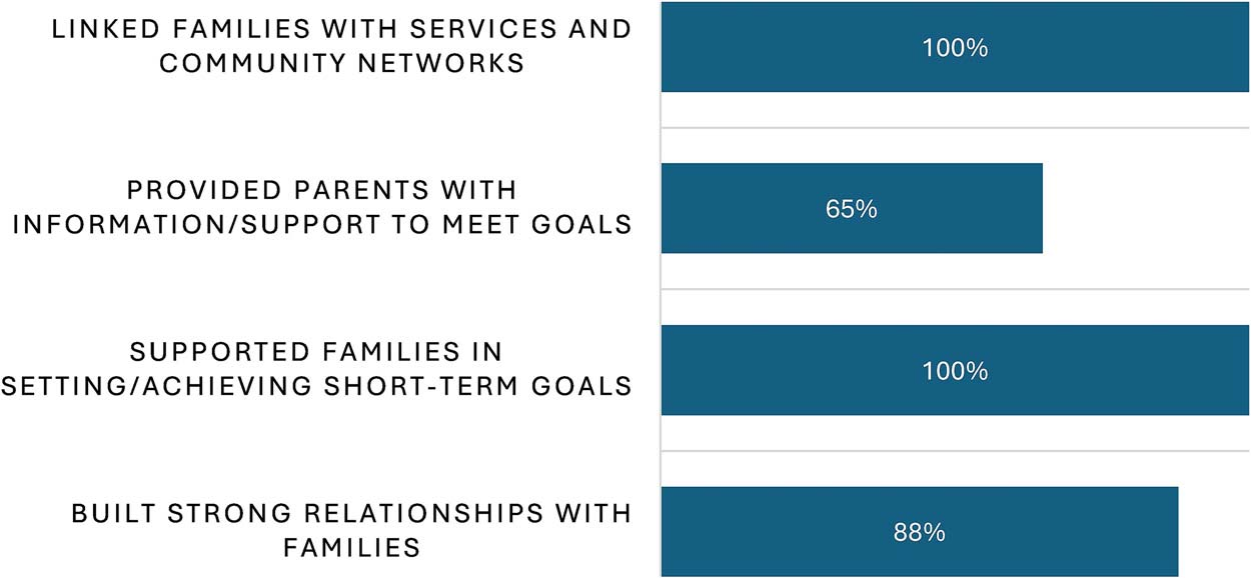
Outcomes reported by CNNs delivering the I2S programme.

The survey findings indicate that the majority of CNNs reported positive outcomes in their work with families. All CNNs stated that they were able to support families in setting and achieving short-term goals, as well as linking them with relevant services and community networks. In addition, 88% reported building strong relationships with families, and 65% reported providing parents with information and support to help them meet their goals (Fig. 3).

Three main themes were identified from the qualitative data.

#### Improved knowledge, skills, and confidence

CNNs consistently reported that participation in the I2S programme enhanced their knowledge, skills, and confidence in working with families. They described learning new strategies to empower and support parents, including the ability to provide culturally sensitive care tailored to individual family contexts:

‘The I2S training has significantly enhanced both my knowledge and skills in delivering the programme to families. I learned different strategies to empower families with skills’.

‘I have learnt more to empower parents and challenge parents to reflect. I have learnt more about different cultures and beliefs and the importance of listening to these beliefs and adapting the support around these’.

For some practitioners, this growth contributed directly to increased job satisfaction, confidence in their professional identity, and a stronger sense of competence in delivering the programme:

‘The I2S training has had a positive impact on my job satisfaction. The training has equipped me with the necessary skills to deliver the programme effectively, increasing my confidence in my role’.

#### Greater enablement to support families

The I2S programme enhanced CNNs’ ability to build meaningful relationships with families, enabling a deeper understanding of family needs and a more proactive approach to care. CNNs described becoming more confident in guiding families towards appropriate services and supports within the community:

‘Identify the need… support and empowering families to meet their goals and link families with the supportive community’.

‘Building relationships with families and getting to understand their needs. Informing and signposting to the community services’.

Participants highlighted the value of the I2S framework in supporting a creative and integrated approach to service delivery. This included facilitating connections between families and local services such as Family Wellbeing Centres (FWCs), arranging nursery visits, and using a wider range of community resources:

‘The I2S role has made me more proactive in finding out the support available and being creative in what support I can offer parents / how to link all services together, i.e., signposting parents to FWC more, carrying out nursery visits’.

Overall, CNNs reported that the training had a transformative effect on their professional practice. It contributed to a more integrated and collaborative way of working, enhanced interdisciplinary communication, and fostered evidence-based decision-making. Several CNNs noted increased enjoyment and fulfilment in their roles, with some expressing a desire to pursue future careers in nursing and/or health visiting. This suggests that the I2S training not only enhanced immediate practice but also supported long-term professional development and career aspirations.

#### Identified areas for programme improvement

Despite the positive impacts reported, CNNs highlighted several areas where the I2S programme could be strengthened to better support both practitioners and families. Key recommendations included the need for enhanced training and resources, particularly in specific practical areas such as behaviour management, toilet training, and sleep. CNNs also expressed a desire for increased access to online resources that families could use independently.

Several practitioners suggested the introduction of ongoing refresher training sessions to help maintain and update skills and knowledge over time. There was also a call for improved support in identifying families who would benefit most from the I2S programme to ensure more targeted and timely intervention.

In terms of data collection, CNNs recommended simplifying and shortening the questionnaires to reduce administrative burden and increase feasibility in busy clinical settings. Finally, additional resources were sought to foster and strengthen multidisciplinary collaboration, recognising the importance of integrated working to deliver holistic care.

These findings offer important direction for further development and refinement of the I2S programme, aimed at improving its overall effectiveness and long-term sustainability.

## Discussion

### Main finding of this study

This study presents the first UK evaluation of the I2S programme, drawing on the perspectives of CNNs delivering the intervention in the London Borough of Brent. CNNs consistently reported that I2S enhanced their skills, confidence, and job satisfaction, enabling them to work more systematically with families and to provide culturally sensitive, relationship-based support. They highlighted improvements in their ability to build trust, set goals with parents, and link families to community resources. Trust between families and practitioners was highlighted as central to success, echoing research on health visiting where continuity and trust underpin parental satisfaction.^24^ These findings suggest that, from the practitioners’ perspective, I2S is supporting more proactive, integrated, and family-centred practice within health visiting teams.

Many also reported feeling more proactive in their role and having a better understanding of how to link families with early years settings and FWCs. This suggests I2S may help develop integrated care pathways, an internationally recognised solution to fragmented early years provision, particularly in disadvantaged communities.^3^^,^^16^^,^^17^ While direct evidence of interdisciplinary collaboration was limited, reflections from CNNs indicate that the processes for more integrated ways of working are emerging.

As discussed earlier, in Brent, school readiness data remains below the London and national averages, and uptake of the 2-year-old offer has historically been low. These challenges are not unique to Brent but are seen in other high-deprivation areas across the UK and internationally.^6^^,^^7^^,^^22^ The I2S programme was introduced to support children most at risk of falling behind and to strengthen existing early years provision. Early findings reported by CNNs suggest that it is helping to do this by offering a structured framework for targeted support delivered by these skill-mix practitioners in health visiting teams.

Areas for improvement included requests for further training (behaviour, toilet training, and sleep), simplified data collection tools, more family resources, and better targeting of eligible families, which are useful practical recommendations for refining the programme.

### What is already known on this topic

Evidence from international home-visiting programmes such as the MECSH programme from Australia and the Family Nurse Partnership (FNP) from the USA, demonstrates that structured early intervention delivered through trusted practitioners can improve parental competence, service engagement, and child development outcomes.^19^^,^^20^^,^^25^ In the UK, evaluations of Sure Start and other early years interventions have similarly emphasised the importance of continuity, trust, and relational working in achieving positive outcomes for families.^8^^,^^14^ However, these programmes typically assess both practitioner and family perspectives, often through longitudinal or controlled designs, which provide stronger evidence of impact.

### What this study adds

This evaluation provides an important first insight into the implementation of the I2S programme in a highly diverse and socioeconomically disadvantaged urban setting. This early evaluation contributes new insight into the role of CNNs in supporting school readiness through a structured, health visiting-embedded programme. Findings suggest that I2S has the potential to strengthen the skill-mix model by empowering CNNs to deliver targeted early intervention within disadvantaged communities. Importantly, I2S appears to provide an enabling framework that enhances CNNs’ sense of professional identity, which may also support workforce development and retention. These findings are encouraging and consistent with international literature on home-visiting interventions, but uniquely highlight the contribution of CNNs, a workforce not widely studied outside the UK.

Preliminary exploratory analyses also suggest patterns between child age and the types of concerns identified by families, such as language development concerns being most prevalent among toddlers and behavioural issues more common in older preschool children. While these patterns are descriptive only, they point to the value of tailoring support to developmental stage and will be explored in more depth in future longitudinal evaluations.

The high response rate (85%) from CNNs ensures that the findings reflect the views of most practitioners actively engaged in the Brent programme at the time. The use of anonymous questionnaires encouraged candid feedback, and the independent analysis by a researcher external to the local implementation team helped reduce potential bias in interpreting responses. Furthermore, the study highlights key areas for programme development and provides practical recommendations for ongoing improvement.

### Limitations of this study

This study has important limitations. The findings are based primarily on CNNs’ self-reported perceptions, which are inherently subjective and may be influenced by social desirability or professional enthusiasm. Without triangulation using parental perspectives or child outcome data, it is not possible to determine the extent to which practitioner-reported outcomes reflect real benefits for families and children. In addition, the bespoke CNN questionnaire was not formally validated, and no baseline data on CNN skills were collected, limiting the ability to attribute perceived improvements directly to programme participation.

The analysis was limited to descriptive statistics due to the small sample size and incomplete outcome data, restricting opportunities for significance testing or baseline–midpoint–endpoint comparisons. While exploratory patterns between child age and presenting concerns were identified, these should be interpreted cautiously. Finally, the Brent context, which is highly diverse and socioeconomically disadvantaged, may limit generalisability, and replication in other areas will be necessary.

Despite these limitations, the evaluation provides valuable early learning and lays the foundation for future longitudinal studies that will incorporate validated child outcome measures (ASQ-3, PaRRiS, and HOME-SF), parental feedback, and more robust statistical analysis to assess the long-term effectiveness and scalability of I2S.

### Recommendations for future research

Building on this initial evaluation, future research should adopt a mixed-methods approach that incorporates multiple data sources to strengthen validity. This includes collecting quantitative child development outcomes using validated tools (such as ASQ-3, PaRRiS, and HOME-SF), alongside qualitative feedback from parents and observations to triangulate data collected through various methods. Establishing baseline measures prior to training and implementation will allow for more rigorous assessment of changes in practitioner skills and family outcomes over time.

Research should also examine scalability across localities, including rural and culturally varied areas, to evaluate the adaptability and sustainability of I2S nationally and internationally.

## Conclusion

This early evaluation of the I2S programme suggests that CNNs feel more confident, skilled, and empowered in their roles, with the structured framework provided by the I2S programme enabling them to deliver more focused, culturally sensitive, and relationship-based support to families. This, in turn, means they are better equipped to support children and families facing adversity, contributing to early identification of developmental concerns, and helping to reduce inequalities in school readiness challenges in areas like Brent.

In Brent, the programme successfully expanded CNN capacity, fostered stronger family relationships, and improved access to community support. Continuity of care emerged as a key strength, reinforcing the value of trust and relational working in early years services. While further research on long-term child outcomes and parent perspectives is needed, this evaluation suggests I2S is a promising model for strengthening early intervention and promoting school readiness.

### Informed Consent Statement

Completion of the survey questionnaire was considered as implied consent to participate in the evaluation. Participants were informed that their participation was voluntary, and responses would be anonymised, and used solely for the purpose of this evaluation. Institutional Review Board Statement: As this was a service evaluation, ethical approval was not required.

## Disclaimer

S.B. completed this work whilst on secondment from the iHV to Western Sydney University. The iHV retains its independent status and this agreement should not be construed as an endorsement by the iHV of Western Sydney University or its actions, including the I2S programme.

## Data Availability

The data presented in this study are available on request from the corresponding author due to organisation privacy restrictions.

## Notes

**Sharin Baldwin,** Senior Health Visitor Research Lead, Adjunct Senior Research Fellow, Postdoctoral Research Fellow

**Liza Azizpoor,** Infant to School Lead Practitioner

**Marzia Keshani,** Health Visitor and MECSH Advisor

**Wendy Sumpton,** MECSH-UK Implementation Consultant

**Marie McLouglin,** Consultant in Public Health

**Kathy Donohoe,** Senior Research Officer

**Lynn Kemp,** Distinguished Professor and Director
